# An Open Letter to Young Anaesthesiologists

**DOI:** 10.5152/tjar.2021.21080

**Published:** 2022-04-01

**Authors:** Shibu Sasidharan, Harpreet Singh Dhillon

**Affiliations:** 1 Department of Anaesthesia and Critical Care, Command Hospital, Chandimandir, Haryana, India; 2 Department of Psychiatry, Command Hospital, Chandimandir, Haryana, India

Throughout almost the whole of 2020, 2021 and at least till this day of 2022 (January 12), the COVID-19 pandemic has impressed drastic implications across all medical specialties, especially anaesthesiology. As a young doctor entering the field of anaesthesiology in such a time of great uncertainty and change, it is only natural why anybody will re-consider the choices you’ve made, especially in the face of your current struggle. As I complete my first few years actively practicing this specialty, I haven’t been more struck by the incredible and selfless examples set by anaesthesiologists the world over, fighting against COVID-19. As their acts of courage and grit in admittedly frightening and difficult situations continue to inspire generations of doctors throughout the world, it has, like for many I know across generations of anaesthesiologists, reaffirmed our decision to become one. The media is replete with examples of anaesthesiologists demonstrating professionalism, tenacity, and innovation while treating patients affected by COVID-19. So if you’re an anesthesiologist or a budding one, you need to pat your back for doing what you do, and I am here to assure you that amid this feeling of anxiety, isolation, and angst, we are all in this together.

However, as we are wearing our caps and are being called into action, we need to counter fear. The way to counter fear and break away from the vicious circle of obsessive and unproductive discussions is to promote attitudes and actions that raise morale. This can be done by the folowing ways:

1. Promote work-life balance among employees.
2. Invest in trust-building.
3. Go beyond “My door is always open.”
4. Give teammates a chance to interact outside the office.
5. Support employee-led initiatives.
6. Do not ignore the power of small gestures.
7. Educate staff members on insurance and medical claims.

When employees feel like their concerns are being listened to, they bring in several benefits as follows:

1. Quicker work turnover rates: Clearing your employee’s concerns can help improve clarity as well as maintain focus to concentrate and complete the tasks on hand.
2. Less distraction: People’s imaginations can often run wild. The gossip and rumor mill might create unwarranted concerns with no truth behind them. This could be a cause of distraction for a number of staff members.
3. Illustrate a caring attitude: Listening and addressing your staff's concerns during the COVID-19 pandemic shows your employees that you care about their health, as well as their safety.

Dear younger colleagues, you will realize how important it is to motivate those around you to have a more forthcoming attitude, contribute with ideas and initiatives, meeting adversity with resourcefulness. That, multiplied by each of you can make significant changes in patient and care for self. Each of us must act as if we had been chosen to trigger a cascade of motivation, confidence, and mutual support. If there are two of us, we will be all the stronger for it. If there are more, the group will become all the more confident and determined. Anesthesiologists value and practice teamwork: we are good at forming teams and know from experience that teamwork and mutual support will provide us with a sense of reassurance and commitment.^1^

Anaesthesiology is a leader in safety, so we are particularly well placed to raise safety levels in the present circumstances. Public health authorities and society at large have engaged in multiple initiatives that allowed us to feel optimistic, in many ways, during the surge of cases. Although anaesthesiologists make up only about 5% of physicians in the United States, anaesthesiology is acknowledged as the leading medical specialty in addressing issues of patient safety.^2^ Why is this so? First, as anaesthesia care became more complex and technological and expanded to include intensive care, it attracted a higher caliber of staff. Clinicians working in anaesthesiology tend to be risk-averse and interested in patient safety because anaesthesia can be dangerous but has no therapeutic benefit of its own.^3^

When the American Medical Association decided to create a National Patient Safety Foundation, it did so in open imitation of the methods and success of the Anaesthesia Patient Safety Foundation.^4^

As you step into this wonderfully skilled physician workforce, let us be inspired by not just the giants, but by every single member of our workforce who continues the legacy of champions of patient safety, patient care, ventilatory support, and palliative care. To the next generation of anaesthesiologists to be, I want to share a tagline of the American Lung Association—“When you can’t breathe, nothing else matters.”

So help them breathe, and join in the joy of healing.
